# HIV-1 transmitted drug resistance mutations among antiretroviral therapy-Naïve individuals in Surabaya, Indonesia

**DOI:** 10.1186/s12981-015-0046-y

**Published:** 2015-02-22

**Authors:** Tomohiro Kotaki, Siti Qamariyah Khairunisa, Adiana Mutamsari Witaningrum, Muhammad Qushai Yunifiar M, Septhia Dwi Sukartiningrum, Muhammad Noor Diansyah, Retno Pudji Rahayu, ᅟ Nasronudin, Masanori Kameoka

**Affiliations:** Institute of Tropical Disease, Indonesia-Japan Collaborative Research Center for Emerging and Re-emerging Infectious Diseases, Airlangga University, Surabaya, Indonesia; Center for Infectious Diseases, Kobe University Graduate School of Medicine, Hyogo, Japan; Faculty of Medicine, Airlangga University, Surabaya, Indonesia; Airlangga University Teaching Hospital, Surabaya, Indonesia; Department of International Health, Kobe University Graduate School of Health Sciences, Hyogo, Japan

**Keywords:** HIV-1, Antiretroviral therapy, Transmitted drug resistance, Indonesia

## Abstract

**Background:**

The emergence of transmitted drug resistance (TDR) compromises the effect of antiretroviral therapy (ART), resulting in treatment failure of human immunodeficiency virus (HIV) disease. Although more than a decade has passed since ART was introduced into Indonesia, information on TDR is limited. Here, a genotypic study of TDR among ART-naïve individuals was conducted in Surabaya, Indonesia.

**Method:**

HIV-1 seropositive participants were recruited from the communities of commercial sex workers and intravenous drug users as well as from the university teaching hospital in Surabaya. Protease (PR) and reverse transcriptase (RT) genes were sequenced in order to conduct HIV-1 subtyping and phylogenetic analysis and to detect TDR. TDR was defined as the presence of at least one surveillance drug resistance mutation on the WHO list or major drug resistance mutations in the International AIDS Society-USA panel.

**Result:**

Fifty two and 47 of the PR and RT genes, respectively, were successfully sequenced in the 58 samples. HIV-1 subtyping revealed that 86.3% (50/58) of the sequenced samples were classified as CRF01_AE, 8.6% as subtype B, 3.4% as B/CRF01_AE, and 1.7% as A/G/CRF01_AE. TDR of PR inhibitors was not detected in this study. In contrast, TDR of RT inhibitors was detected in 4.3% (2/47) of samples. In addition, minor drug resistance mutations were detected in 98.1% (51/52) and 12.8% (6/47) of PR and RT genes, respectively.

**Conclusion:**

This study clarified the predominance of the CRF01_AE strain in Surabaya, Indonesia. The prevalence of TDR was below 5%, indicating that the currently available first-line regimen is still effective in Surabaya. However, the prevalence might be underestimated since we detected only major population of HIV-1 in individuals. Therefore, continuous surveillance is required in order to detect the emergence of TDR in the early phase.

**Electronic supplementary material:**

The online version of this article (doi:10.1186/s12981-015-0046-y) contains supplementary material, which is available to authorized users.

## Backgrounds

Antiretroviral therapy (ART) achieved the reduction of viral transmission, morbidity and mortality associated with human immunodeficiency virus (HIV) disease. However, the emergence of transmitted drug resistance (TDR) as a consequence of ART expansion represents a serious public health problem because TDR affects the treatment efficacy and clinical outcome [1,2]. Although the prevalence of TDR in resource-limited countries is currently <5% [3], it is expected to increase with ART expansion. TDR is a permanent challenge for HIV disease control.

HIV-1 is subdivided into four groups, M (major), O (outlying), N (new or non-M, non-O), and P. Group M accounts for the majority of HIV-1 infections. The viruses in group M are further classified into subtypes, circulating recombinant forms (CRFs) and unique recombinant forms (URFs), which are prevalent in specific geographic regions. While subtype B of HIV-1 is the predominant subtype in the Americas, Europe, and Australia, there is a growing epidemic of non-B subtypes and CRFs in Africa and Asia [4]. CRF01_AE is the major CRF prevalent throughout Southeast Asian countries including Indonesia [4]. Although surveillance studies on TDR have been conducted in Southeast Asian countries where CRF01_AE is prevalent, information on TDR as well as on CRF01_AE viruses is still limited [3,5].

In Indonesia, the estimated number of people living with HIV has been increasing and reached 610,000 in 2012, even though that of other Southeast Asian countries is stable or in decline [6]. Accordingly, it was estimated that the number of people eligible for ART, whose CD4+ T-cell count was below 500 cells/mm^3^ (WHO criteria), would reach 510,000 in 2013 [6]. Even though the Indonesian government launched an ART expansion program in 2004 [7], the coverage rate among patients in need of ART was less than 18% until 2010 [6]. However, it increased markedly to 40% in 2011 according to the Ministry of Health, Mathematic Model of HIV Epidemic in Indonesia [8]. The number of patients with access to ART is therefore increasing in Indonesia.

The first-line regimen of ART recommended in Indonesia is a combination of two nucleoside reverse-transcriptase inhibitors (NRTIs) and a non-nucleoside reverse-transcriptase inhibitor (NNRTI) [5]. Lamivudine (3TC), zidovudine (AZT), tenofovir (TDF), nevirapine (NVP) and efavirenz (EFV) are commonly used. For patients with virological failure or adverse effects, ritonavir-boosted protease inhibitors (PIs) in combination with two NRTIs are recommended as the second-line regimen [5]. Other drugs, including didanosine (ddI), etravirine (ETR) and rilpivirine (RPV), are uncommon in Indonesia.

Although ART is successful in Indonesia, the emergence of drug resistance has been reported among treatment-failure patients [9]. After a decade of ART expansion in Indonesia, the emergence of TDR is inevitable. However, there are limited data on TDR among ART-naïve patients in Indonesia. It is important to monitor the prevalence of TDR in countries where the drug options are limited. In this report, a genotypic study of TDR among ART-naïve patients was conducted in Surabaya, Indonesia.

## Results

### Demographic data of the study subjects

Peripheral blood samples were collected from the participants from the communities of commercial sex workers (CSWs) and intravenous drug users (IDUs) as well as from the university teaching hospital in Surabaya. All participants were confirmed to be ART naïve at interview and/or from medical records. RNA and DNA were extracted from plasma and peripheral blood mononuclear cells (PBMC), respectively, isolated from peripheral blood samples. If a viral gene fragment failed to be amplified from the cDNA even after multiple attempts, it was amplified instead from DNA. In order to examine the genomic fragment of the major viral population in a sample, PCR products amplified at the end-point dilution of DNA templates were subjected to sequencing analysis.

As a result, 52 and 47 of the protease (PR) and reverse transcriptase (RT) genes, respectively, were successfully sequenced in the 58 samples. Of these, 13 and 22 of the PR and RT genes, respectively, were derived from DNA. The demographic data of the 58 individuals are shown in Table 1, along with the results of viral subtyping described below. Mean age was 33.2 years old (range 17–51). There was no significant difference in age and subtype among study participants. Detailed patient information including age, sample collection date and transmission route is shown in Additional file 1.

**Table 1:** Demographic characteristics, viral subtypes and time after infection of study subjects*

	**All (n = 58)**	**CSW community (n = 22)**	**IDU community (n = 16)**	**Hospital (n = 20)**
Mean age (years old)	32.2	30.3	33.9	33.0
Gender				
Male	26 (44.8%)**	0 (0%)	15 (93.8%)	11 (55.0%)
Female	32 (55.2%)	22 (100%)	1 (6.2%)	9 (45.0%)
Viral subtype				
CRF01_AE	50 (86.3%)	20 (91.0%)	12 (75.0%)	18 (90.0%)
Subtype B	5 (8.6%)	0 (0%)	3 (18.8%)	2 (10.0%)
B/CRF01_AE recombinant	2 (3.4%)	1 (4.5%)	1 (6.2%)	0 (0%)
A/G/CRF01_AE recombinant	1 (1.7%)	1 (4.5%)	0 (0%)	0 (0%)

*There was no significant difference among study participants from the communities of CSWs and IDUs, and from Airlangga University Teaching Hospital in terms of mean age and viral subtype. Gender was statistically different because we aimed to recruit female CSW.

**The proportion (%) of the number of individuals in a question item is shown in parentheses.

### HIV-1 subtyping and phylogenetic analysis

**Figure 1:**
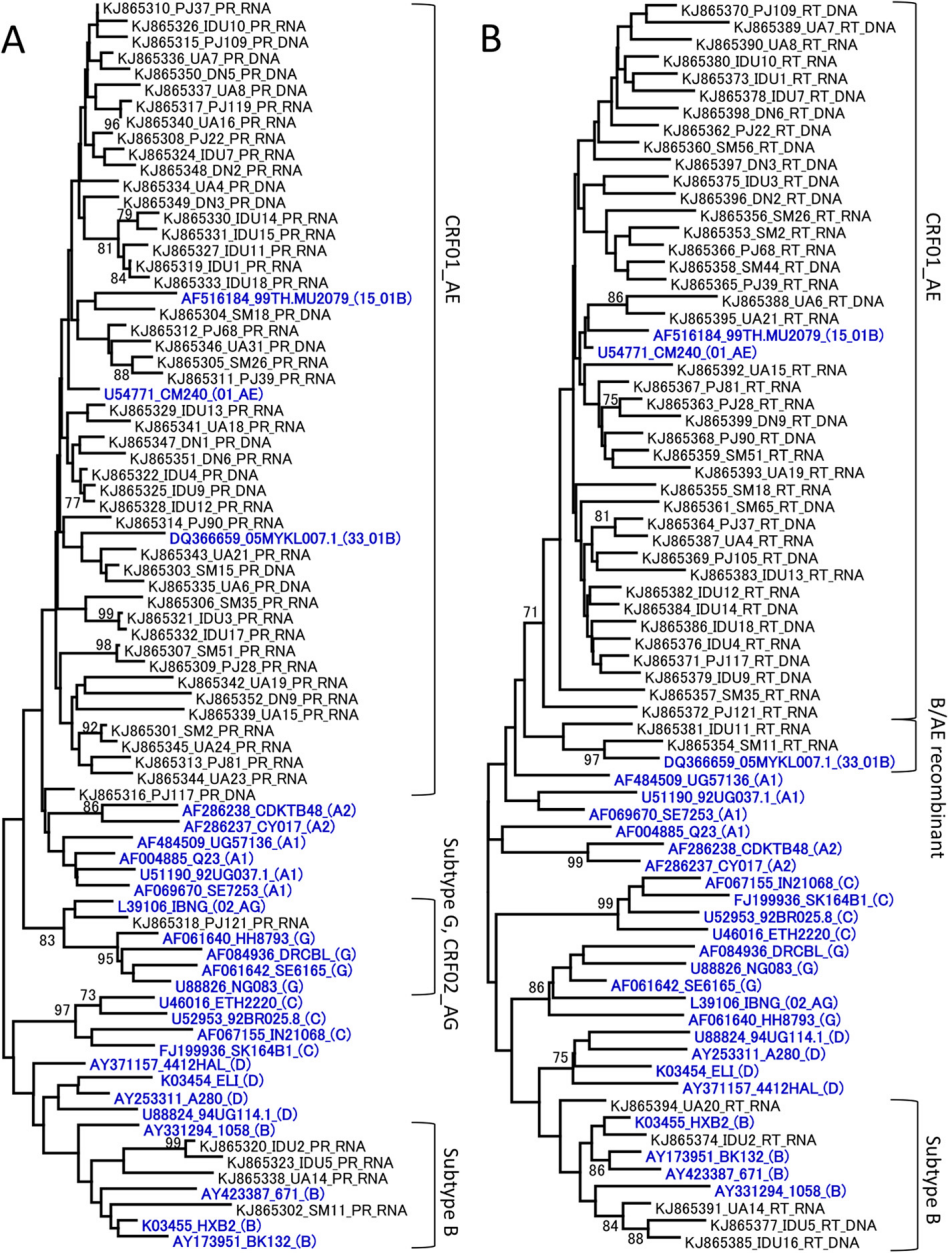
**Neighbor-joining phylogenetic trees of PR and RT gene sequences.** Phylogenetic trees were constructed for newly sequenced HIV-1 PR **(A)** and RT **(B)** genes along with the corresponding viral gene of reference HIV-1 strains, representing subtype A1, subtype A2, subtype B, subtype C, subtype D, subtype G, CRF01_AE, CRF02_AG, CRF15_01B and CRF33_01B. The reference strains of the HIV-1 subtype were shown in blue and bold. The sequence codes are presented as the GenBank accession number, patient ID or name of reference strain, and the subtype or CRF of reference strain (shown in parentheses) in order. DNA or RNA following the patient ID indicates the origin of the viral sequence, i.e., DNA extracted from PBMC or RNA extracted from a plasma sample, respectively. Bootstrap values are shown when the values are >70.

HIV-1 subtyping was carried out for PR and RT gene sequences separately using recombinant identification program (RIP). Most PR and RT gene sequences were classified as CRF01_AE or subtype B. However, two RT gene sequences derived from SM11 and IDU11 were classified as B/CRF01_AE, and PR and RT gene sequences derived from PJ121 were G/CRF01_AE and A/G/CRF01_AE, respectively (Additional file 1). Phylogenetic trees of PR and RT gene sequences are shown in Figure 1. HIV-1 subtyping by phylogenetic analysis was consistent with RIP results, except for PJ121. With regard to the samples from PJ121, for the PR gene, phylogenetic analysis showed that it was clustered with subtype G and CRF02_AG, while RIP showed that it was G/CRF01_AE. For the RT gene, phylogenetic analysis showed its relevance to CRF01_AE, whereas RIP showed that it was A/G/CRF01_AE. Taken together, 50 samples (86.3%) were classified as CRF01_AE, 5 samples (8.6%) as subtype B, 2 samples (3.4%) as B/CRF01_AE and a sample (1.7%) derived from PJ121 as A/G/CRF01. In addition, jumping profile Hidden Markov Models (jpHMM) analysis revealed that viral gene fragments derived from SM11 and IDU11 (B/CRF01_AE) had their breakpoints at 2884–2883 and 2889–2909 (HXB2 position), respectively, which were similar to that of CRF33_01B reference strains (2847–2884) (Additional file 2). The breakpoint of the viral gene fragment from PJ121 was not similar to that of the CRFs registered in the Los Alamos HIV sequence database (http://www.hiv.lanl.gov/content/sequence/HIV/CRFs/CRFs.html).

### Genotypic analysis of drug resistance for PR gene

In this study, TDR was defined as the presence of at least one major drug resistance mutation listed in the international AIDS Society (IAS)-USA panel or surveillance drug resistance mutation in world health organization (WHO) surveillance list [10,11]. According to the lists, TDR was not detected in PR gene sequences. However, minor drug resistance mutations listed in the IAS-USA panel were detected in 98.1% (51/52) of the study subjects, shown in Table 2 [10]: L10I/V, G16E, K20R, L33F, M36I/L, I62V, L63P, I64V, H69K/R, V77I, V82I, L89M/I and I93L. Each CRF01_AE strain possessed at least 3 and up to 8 mutations. M36I/L, H69K/R and L89M/I were highly conserved in the CRF01_AE strain, at rates of 95.7% (45/47), 100% and 100%, respectively (Table 2). These mutations were known as natural polymorphism among CRF01_AE [12].

**Table 2:** Appearance of minor drug resistance mutations in PR gene*

**Mutation**	**Frequency (%)**			
	**All (n = 52)**	**CRF01_AE (n = 47)**	**Subtype B (n = 4)**	**G/CRF01_AE (n = 1)**
L10I/V	11 (21.2)	10 (21.3)	1 (25.0)	0 (0)
G16E	16 (30.8)	15 (31.9)	1 (25.0)	0 (0)
K20R	19 (36.5)	19 (40.4)	0 (0)	0 (0)
L33F	7 (13.5)	7 (14.9)	0 (0)	0 (0)
M36I/L	46 (88.5)	45 (95.7)	0 (0)	1 (100)
I62V	6 (11.5)	6 (12.8)	0 (0)	0 (0)
L63P	5 (9.6)	4 (8.5)	0 (0)	1 (100)
I64V	3 (5.8)	2 (4.3)	1 (25.0)	0 (0)
H69K/R	48 (92.3)	47 (100)	0 (0)	1 (100)
V77I	12 (23.1)	10 (21.3)	2 (50.0)	0 (0)
V82I	3 (5.8)	3 (6.4)	0 (0)	0 (0)
L89M/I	48 (92.3)	47 (100)	0 (0)	1 (100)
I93L	15 (28.8)	12 (25.5)	3 (75.0)	0 (0)

*Mutations were detected manually according to the International AIDS Society-USA panel.

### Genotypic analysis of drug resistance for RT gene

Two of 47 samples (4.3%) possessed TDR in RT gene sequences. The RT gene fragment from SM18 contained a K101E mutation, which is a major drug resistance mutation to NNRTI; ETR and RPV [10]. Interestingly, the sample from UA6 contained 6 major drug resistance mutations: D67N, K70R, M184V, T215F, K219Q, conferring resistant to almost all NRTI (except ddI), and K103N conferring resistance to NNRTI; NVP and EFV [10]. RT gene fragment of UA6 was amplified from undiluted DNA (because of the failure of PCR from diluted DNA), indicating that minor population of HIV also possessed multiple TDR. The failure of PCR using RNA might be due to poor sample storage condition. Other than TDR, four minor drug resistance mutations (A98G, E138G/A, V179D), which confer resistance to ETR and/or RPV, were detected among 6 samples of CRF01_AE (12.8%: 6/47) [10]. The data are summarized in Table 3.

**Table 3:** Demographic characteristics of 6 individuals infected with HIV-1 with drug resistance mutation to RT inhibitors*

**ID**	**Group**	**Subtype**	**Drug resistance mutation**		
			**NNRTI**	**NRTI**	**Resistance**
SM18	CSW	CRF01_AE	K101E**E138G		RPV*** ETR
UA6	Hospital	CRF01_AE	**K103N** A98G	**D67N K70R, M184V T215F K219Q**	**3TC, AZT, TDF, NVP, EFV, ABC, d4T, FTC,** ETR, RPV
IDU9	IDU	CRF01_AE	E138A		RPV, ETR
UA7	Hospital	CRF01_AE	E138A		RPV, ETR
UA15	Hospital	CRF01_AE	V179D		RPV, ETR
DN6	Hospital	CRF01_AE	E138G		RPV, ETR

*HIV-1 drug resistance mutations were detected according to the International AIDS Society-USA panel and WHO surveillance list.

**Major drug resistance mutations are shown in bold.

***Highly resistant drugs are shown in bold.

## Discussion

Here we report the circulating HIV-1 subtype and the prevalence of TDR among 58 HIV-1-infected drug-naïve individuals in Surabaya, Indonesia. Of 58 sequenced samples, 50 (86.3%) were classified as CRF01_AE, 5 (8.6%) as subtype B, 2 (3.4%) as B/CRF01_AE, and 1 (1.7%) as A/G/CRF01_AE. These results are consistent with a previous report on the predominance of CRF01_AE in Southeast Asian countries [4]. According to the phylogenetic tree of RT gene and jpHMM analysis, B/CRF01_AE recombinants in Surabaya were closely related to CRF33_01B, which was currently reported in Indonesia [13]. The viral gene derived from PJ121, an A/G/CRF01_AE recombinant, seems a URF because its breakpoint and recombination pattern had not been reported as of December 2014, in the Los Alamos HIV sequence database. The HIV gene fragment analyzed in the present study was not informative enough to determine actual CRFs. Further sequence analysis of different regions must therefore be carried out.

Genotypic analysis of drug resistance revealed no evidence of the circulation of TDR against PIs in Surabaya. This was not surprising because access to PIs is limited in Surabaya, even though PI is recommended as the second-line regimen. Interestingly, each CRF01_AE strain possessed 3 to 8 minor drug resistance mutations based on the IAS-USA panel [10]. Although these mutations were considered natural polymorphisms of CRF01_AE, the accumulation of minor mutations may affect the efficacy of PIs. Further studies are necessary to clarify their impact on the susceptibility of PIs.

In contrast, the prevalence of TDR against RT inhibitors was 4.3% (2/47). This was consistent with a previous report that TDR in Asian countries was <5%, but is gradually increasing [3]. The sample from SM18 contained a K101E mutation conferring resistance to ETR and RPV [10]. However, the emergence of K101E is probably not associated with ART expansion in Indonesia because ETR and RPV are not commonly used. It is considered that K101E appeared as a natural polymorphism in Indonesian CRF01_AE. In contrast, the RT gene fragment derived from UA6 possessed 6 major mutations and 1 minor mutation. The virus in UA6 is considered to be resistant to the common first-line regimen in Indonesia (3TC, AZT, TDF, NVP and EFV). The patient UA6 was probably infected with HIV-1 by heterosexual intercourse (Additional file 1), indicating that the HIV-1 with multiple TDR had enough growth fitness for sexual transmission. A multiple drug resistant HIV was confirmed among drug-naïve HIV patient in Surabaya.

Four minor mutations (A98G, E138G/A, V179D) conferring resistance to ETR and/or RPV were detected among 6 samples. However, they are considered natural polymorphisms because these mutations are excluded from the WHO list [11], and ETR and RPV are not commonly used in Indonesia.

In the WHO TDR surveillance guideline, the prevalence of TDR is categorized into three groups: <5% (low level), 5–15% (medium level) and >15% (high level) [14,15]. The <5% level is the desired threshold for any country scaling up ART. According to this guideline, the currently recommended first- and second-line regimens are appropriate in Surabaya, Indonesia. However, there were several limitations in the design of this study. Our sample collection might have been biased because participants were recruited from CSW and IDU communities. In addition, although the WHO guidelines aimed to recruit recently infected individuals (below 25 years of age, CD4^+^ T-cell counts above 500 cells/mm^3^) [15], these were not used in this study for practical reasons. A previous report indicated that certain drug resistance mutations would revert to the wild type in the absence of selective pressure by ART [16]. Therefore, it is probable that our data may underestimate the prevalence of TDR. However, the TDR including most thymidine analogue mutations (M41L, D67N, K70R, L210W, T215 revertant but not T215Y/F and K219Q) and K103N, which were stable in the absence of ART, were rarely detected in our study subjects even though they have been frequently detected among treatment-failure patients in Indonesia [9,16]. Therefore, it was estimated that the prevalence of TDR among the population recently infected with HIV-1 is not high. It is helpful for the future ART strategy to clarify the prevalence of TDR among patients who are about to start ART, even if the study subjects are biased and may not reflect recent infections. Other than the quality of samples, direct sequencing might cause underestimation, as previously reported [17,18]. Considering these limitations, the data must be evaluated carefully.

## Conclusion

The prevalence of TDR among ART-naïve patients in Surabaya was <5%, which is categorized as low by WHO. However, additional or continuous surveillance must be conducted because this study had several limitations, and the prevalence might be underestimated and biased. The low TDR observed in this study calls for continuous monitoring of TDR in order to detect the emergence of TDR in the early phase.

## Materials and methods

### Ethics statement

This study was conducted with approval from the ethics committee of the Airlangga University (permission number: 25-995/UN3.14/PPd/2013) and the medical ethics committee at Kobe University Graduate School of Medicine (permission number: 784), and written informed consent was obtained from study participants.

### Participant recruitment and sample collection

Participants were recruited from the communities of CSWs and IDUs as well as from the university teaching hospital in Surabaya. The CSW study subjects were the same as in a previous report [19]. All participants were confirmed to be ART naïve at interview and/or from medical records. Samples were collected between October 2012 and April 2014. Ten milliliters of ethylenediaminetetraacetic acid anti-coagulated peripheral blood was collected from each participant. Plasma was then isolated from peripheral blood samples. In addition, peripheral blood mononuclear cells (PBMC) were isolated by density gradient centrifugation using Histopaque 1077 (Sigma-Aldrich, St. Louis, MO, USA). RNA and DNA were extracted from plasma and PBMC using the QIAamp Viral RNA Mini kit (Qiagen, Hilden, Germany) and the GenElute Mammalian Genomic DNA Miniprep kit (Sigma-Aldrich), respectively.

### Amplification of HIV-1 genomic fragment and sequence analysis

Viral RNA was reverse transcribed to cDNA using the SuperScript III First-Stand Synthesis kit (Invitrogen, Carlsbad, CA, USA). Generated cDNA was subjected to amplification of the viral gene for both the PR and RT encoding regions in a separate reaction by nested PCR using Ex Taq (Takara Bio, Shiga, Japan). Primer information is shown in Additional file 3. If a viral gene fragment failed to be amplified from the cDNA even after multiple attempts, it was amplified instead from DNA extracted from PBMC. In order to examine the genomic fragment of the major viral population in a sample, PCR products amplified at the end-point dilution of DNA templates were subjected to sequencing analysis.

Sequencing analysis of the amplified fragment was performed using the BigDye Terminator v3.1 Cycle Sequencing kit with an ABI PRISM 3500 × l genetic analyzer (Applied Biosystems, Foster City, CA, USA). Data were assembled and aligned using Genetyx ver. 10 software (Genetyx, Tokyo, Japan). Nearly the full length of the PR gene [(280 bp; corresponding to nucleotides 2262 to 2541 of a HIV-1 reference strain, HXB2 (GenBank accession no. K03455)] and part of the RT gene (762 bp; nucleotides 2550 to 3311) were sequenced and subjected to subsequent analysis.

### HIV-1 subtyping and phylogenetic analysis

Obtained sequence data were subjected to HIV-1 subtyping using the RIP, available at the website of the HIV sequence database (www.hiv.lanl.gov/). In addition, neighbor-joining (NJ) trees with a Kimura two-parameter model were constructed using MEGA5.2 software [20-22]. Bootstrap values (1,000 replicates) for relevant nodes were reported on a representative tree [23]. If one of the PR and RT genes are failed to be sequenced, the subtype was assigned based on the other gene. In addition, breakpoints of the recombinant forms were determined by using the jpHMM (http://jphmm.gobics.de/) [24]. The nucleotide sequences of these PR and RT genes have been deposited in the GenBank database under accession numbers KJ865301-KJ865352 and KJ865353-KJ865399.

### Detection of drug resistance mutations

HIV-1 TDRs were detected manually according to the IAS-USA panel and WHO surveillance list [10,11]. In this study, TDR in a patient was defined as the presence of at least one major drug resistance mutation listed in the IAS-USA panel or surveillance drug resistance mutation in WHO surveillance list. In addition to TDR, minor drug resistance mutations in the IAS-USA panel were detected as well [10].

### Statistical analysis

Statistical analysis was performed using Fisher’s exact test for categorical variables. The P value was calculated using the program available at the web site, http://aoki2.si.gunma-u.ac.jp/exact/fisher/getpar.html. P values ≤0.05 were considered to be significant.

## Additional files

Additional file 1:
**Detailed patient information of each participant.**
Additional file 2:
**Subtyping analysis of the recombinant HIV-1 strains using jpHMM A-C).** jpHMM results of SM11, PJ121 and IDU11. Numbers in this figure are based on HXB2 numbering. Posterior probability values are indicated on the y axis and nucleotide positions based on the HXB2 sequence are shown on the x axis. Additional file 3:
**Primer information.**

## Abbreviations

ART: Antiretroviral therapy
HIV: Human immunodeficiency virus
TDR: Transmitted drug resistance
CRF: Circulating recombinant form
URF: Unique recombinant form
NRTI: Nucleoside reverse-transcriptase inhibitor
NNRTI: Non-nucleoside reverse-transcriptase inhibitor
3TC: Lamivudine
AZT: Zidovudine
TDF: Tenofovir
NVP: Nevirapine
EFV: Efavirenz
PI: Protease inhibitor
ddI: Didanosine
ETR: Etravirine
RPV: Rilpivirine
CSW: Commercial sex worker
IDU: Intravenous drug user
PBMC: Peripheral blood mononuclear cells
PR: Protease
RT: Reverse transcriptase
RIP: Recombinant Identification Program
jpHMM: Jumping profile Hidden Markov Models
IAS: International AIDS Society
WHO: World Health Organization
NJ: Neighbor-joining
