# Supplementary material for: HIV-1 transmitted drug resistance mutations among antiretroviral therapy-Naïve individuals in Surabaya, Indonesia
Source: AIDS Res Ther. 2015 Feb 22;12:5. doi: 10.1186/s12981-015-0046-y (PMC4336490; doi:10.1186/s12981-015-0046-y)
Supplement: Additional file 1: — Detailed patient information of each participant. [file 12981_2015_46_MOESM1_ESM.pdf]

Additional file 1

Detailed patient information of each participant

						PR (n=52)		RT (n=47)		
Patient ID	Group	Date of sample collection	Age	Gender	Likely transmission route	Subtype*	GenBank accession number	Subtype*	GenBank accession number	Assigned subtype
SM2	CSW	Oct-12	26	Female	Heterosexual	CRF01_AE	KJ865301	CRF01_AE	KJ865353	CRF01_AE
SM11	CSW	Oct-12	-	Female	Heterosexual	Subtype B	KJ865302	B/ CRF01_AE	KJ865354	B/CRF01_AE
SM15	CSW	Oct-12	29	Female	Heterosexual	CRF01_AE	KJ865303	-	-	CRF01_AE
SM18	CSW	Oct-12	23	Female	Heterosexual	CRF01_AE	KJ865304	CRF01_AE	KJ865355	CRF01_AE
SM26	CSW	Oct-12	30	Female	Heterosexual	CRF01_AE	KJ865305	CRF01_AE	KJ865356	CRF01_AE
SM35	CSW	Oct-12	29	Female	Heterosexual	CRF01_AE	KJ865306	CRF01_AE	KJ865357	CRF01_AE
SM44	CSW	Oct-12	25	Female	Heterosexual	-	-	CRF01_AE	KJ865358	CRF01_AE
SM51	CSW	Nov-12	28	Female	Heterosexual	CRF01_AE	KJ865307	CRF01_AE	KJ865359	CRF01_AE
SM56	CSW	Nov-12	31	Female	Heterosexual	-	-	CRF01_AE	KJ865360	CRF01_AE
SM65	CSW	Nov-12	29	Female	Heterosexual	-	-	CRF01_AE	KJ865361	CRF01_AE
PJ22	CSW	Nov-12	27	Female	Heterosexual	CRF01_AE	KJ865308	CRF01_AE	KJ865362	CRF01_AE
PJ28	CSW	Nov-12	41	Female	Heterosexual	CRF01_AE	KJ865309	CRF01_AE	KJ865363	CRF01_AE
PJ37	CSW	Nov-12	22	Female	Heterosexual	CRF01_AE	KJ865310	CRF01_AE	KJ865364	CRF01_AE
PJ39	CSW	Nov-12	35	Female	Heterosexual	CRF01_AE	KJ865311	CRF01_AE	KJ865365	CRF01_AE
PJ68	CSW	Nov-12	27	Female	Heterosexual	CRF01_AE	KJ865312	CRF01_AE	KJ865366	CRF01_AE
PJ81	CSW	Nov-12	40	Female	Heterosexual	CRF01_AE	KJ865313	CRF01_AE	KJ865367	CRF01_AE

PJ90	CSW	Dec-12	23	Female	Heterosexual	CRF01_AE	KJ865314	CRF01_AE	KJ865368	CRF01_AE
PJ105	CSW	Dec-12	41	Female	Heterosexual	-	-	CRF01_AE	KJ865369	CRF01_AE
PJ109	CSW	Dec-12	42	Female	Heterosexual	CRF01_AE	KJ865315	CRF01_AE	KJ865370	CRF01_AE
PJ117	CSW	Dec-12	27	Female	Heterosexual	CRF01_AE	KJ865316	CRF01_AE	KJ865371	CRF01_AE
PJ119	CSW	Dec-12	35	Female	Heterosexual	CRF01_AE	KJ865317	-	-	CRF01_AE
PJ121	CSW	Dec-12	26	Female	Heterosexual	G/ CRF01_AE	KJ865318	A/G/ CRF01_AE	KJ865372	A/G/CRF01_AE
IDU1	IDU	Apr-13	31	Male	IDU	CRF01_AE	KJ865319	CRF01_AE	KJ865373	CRF01_AE
IDU2	IDU	Apr-13	37	Male	IDU	Subtype B	KJ865320	Subtype B	KJ865374	Subtype B
IDU3	IDU	Apr-13	41	Female	IDU	CRF01_AE	KJ865321	CRF01_AE	KJ865375	CRF01_AE
IDU4	IDU	Jun-13	33	Male	IDU	CRF01_AE	KJ865322	CRF01_AE	KJ865376	CRF01_AE
IDU5	IDU	Jun-13	36	Male	IDU	Subtype B	KJ865323	Subtype B	KJ865377	Subtype B
IDU7	IDU	Jun-13	33	Male	IDU	CRF01_AE	KJ865324	CRF01_AE	KJ865378	CRF01_AE
IDU9	IDU	Jun-13	33	Male	IDU	CRF01_AE	KJ865325	CRF01_AE	KJ865379	CRF01_AE
IDU10	IDU	Jun-13	30	Male	IDU	CRF01_AE	KJ865326	CRF01_AE	KJ865380	CRF01_AE
IDU11	IDU	Jun-13	24	Male	IDU	CRF01_AE	KJ865327	B/ CRF01_AE	KJ865381	B/CRF01_AE
IDU12	IDU	Jun-13	37	Male	IDU	CRF01_AE	KJ865328	CRF01_AE	KJ865382	CRF01_AE
IDU13	IDU	Jun-13	45	Male	IDU	CRF01_AE	KJ865329	CRF01_AE	KJ865383	CRF01_AE
IDU14	IDU	Jun-13	35	Male	IDU	CRF01_AE	KJ865330	CRF01_AE	KJ865384	CRF01_AE
IDU15	IDU	Jun-13	35	Male	IDU	CRF01_AE	KJ865331	-	-	CRF01_AE
IDU16	IDU	Jun-13	32	Male	IDU	-	-	Subtype B	KJ865385	Subtype B
IDU17	IDU	Jun-13	32	Male	IDU	CRF01_AE	KJ865332	-	-	CRF01_AE

IDU18	IDU	Jun-13	29	Male	IDU	CRF01_AE	KJ865333	CRF01_AE	KJ865386	CRF01_AE
UA4	hospital	Oct-12	-	Female	heterosexual	CRF01_AE	KJ865334	CRF01_AE	KJ865387	CRF01_AE
UA6	hospital	Oct-12	43	Male	heterosexual	CRF01_AE	KJ865335	CRF01_AE	KJ865388	CRF01_AE
UA7	hospital	Jan-13	-	Male	heterosexual	CRF01_AE	KJ865336	CRF01_AE	KJ865389	CRF01_AE
UA8	hospital	Feb-13	25	Female	heterosexual	CRF01_AE	KJ865337	CRF01_AE	KJ865390	CRF01_AE
UA14	hospital	Oct-13	19	Male	heterosexual	Subtype B	KJ865338	Subtype B	KJ865391	Subtype B
UA15	hospital	Oct-13	36	Female	IDU	CRF01_AE	KJ865339	CRF01_AE	KJ865392	CRF01_AE
UA16	hospital	Oct-13	39	Male	heterosexual	CRF01_AE	KJ865340	-	-	CRF01_AE
UA18	hospital	Oct-13	39	Female	IDU	CRF01_AE	KJ865341	-	-	CRF01_AE
UA19	hospital	Oct-13	31	Male	IDU	CRF01_AE	KJ865342	CRF01_AE	KJ865393	CRF01_AE
UA20	hospital	Oct-13	40	Female	heterosexual	-	-	Subtype B	KJ865394	Subtype B
UA21	hospital	Nov-13	24	Male	heterosexual	CRF01_AE	KJ865343	CRF01_AE	KJ865395	CRF01_AE
UA23	hospital	Nov-13	28	Male	heterosexual	CRF01_AE	KJ865344	-	-	CRF01_AE
UA24	hospital	Nov-13	34	Male	heterosexual	CRF01_AE	KJ865345	-	-	CRF01_AE
UA31	hospital	Apr-14	32	Female	heterosexual	CRF01_AE	KJ865346	-	-	CRF01_AE
DN1	hospital	Mar-14	34	Male	heterosexual	CRF01_AE	KJ865347	-	-	CRF01_AE
DN2	hospital	Mar-14	29	Male	heterosexual	CRF01_AE	KJ865348	CRF01_AE	KJ865396	CRF01_AE
DN3	hospital	Mar-14	17	Male	heterosexual	CRF01_AE	KJ865349	CRF01_AE	KJ865397	CRF01_AE
DN5	hospital	Apr-14	40	Female	heterosexual	CRF01_AE	KJ865350	-	-	CRF01_AE
DN6	hospital	Apr-14	32	Female	heterosexual	CRF01_AE	KJ865351	CRF01_AE	KJ865398	CRF01_AE
DN9	hospital	Apr-14	52	Female	heterosexual	CRF01_AE	KJ865352	CRF01_AE	KJ865399	CRF01_AE

* Subtyping was performed using recombinant identification program.
