# Supplementary material for: HIV-1 transmitted drug resistance mutations among antiretroviral therapy-Naïve individuals in Surabaya, Indonesia
Source: AIDS Res Ther. 2015 Feb 22;12:5. doi: 10.1186/s12981-015-0046-y (PMC4336490; doi:10.1186/s12981-015-0046-y)

A SM11 RT RNA

Fragment Start Position	Uncertainty Region Start - End	Break Point Interval Start - End	Fragment End position	Fragment Subtype
2550	-	2844-2883	2873	Subtype B
2874	-	-	3311	CRF01_AE

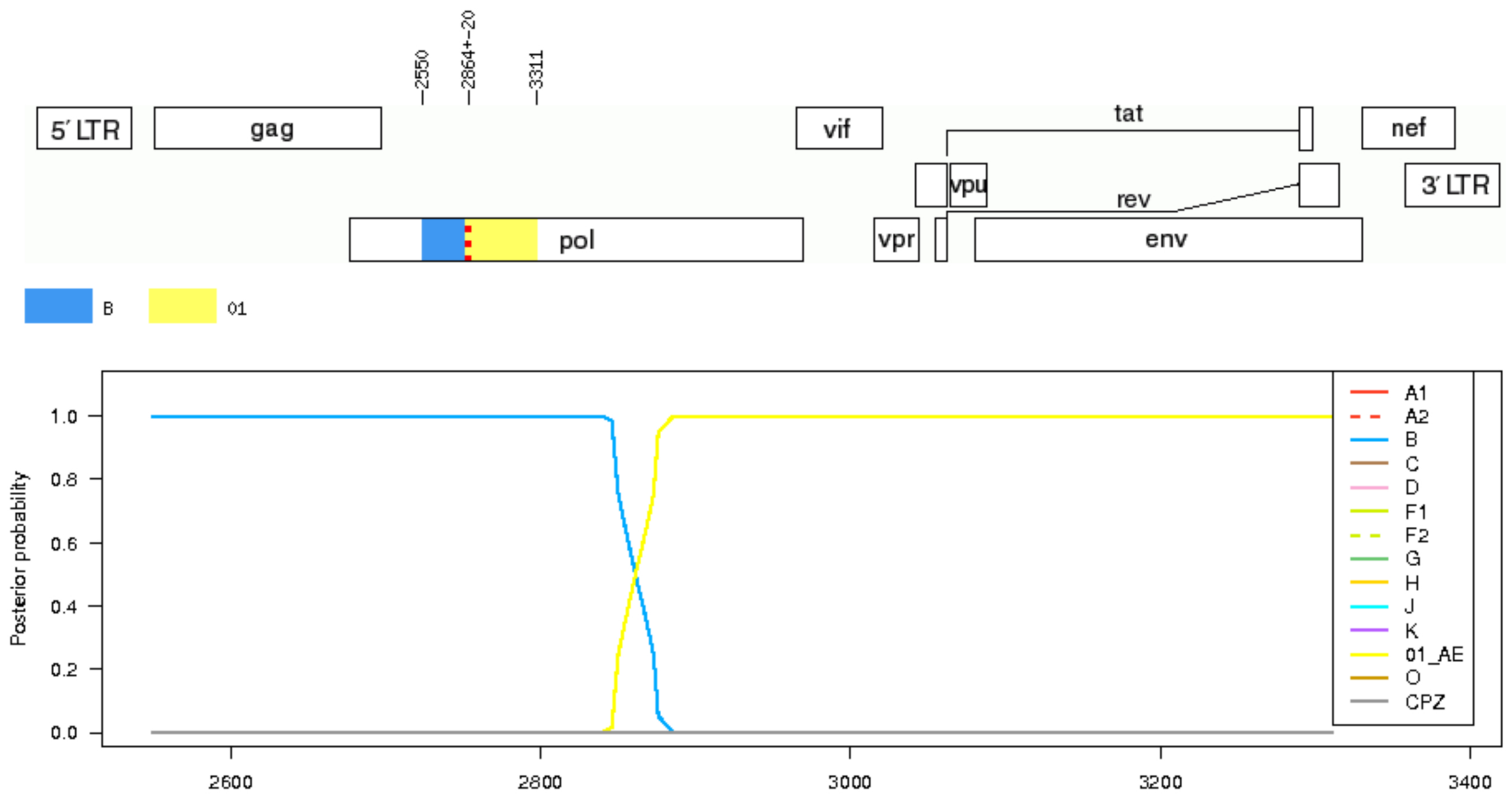

B PJ121 RT RNA

Fragment Start Position	Uncertainty Region Start - End	Break Point Interval Start - End	Fragment End position	Fragment Subtype
2550	2689-3034	-	3034	Subtype A1
3035	3035-3038	3195-3206	3195	Subtype G
3196	-	-	3311	CRF01_AE

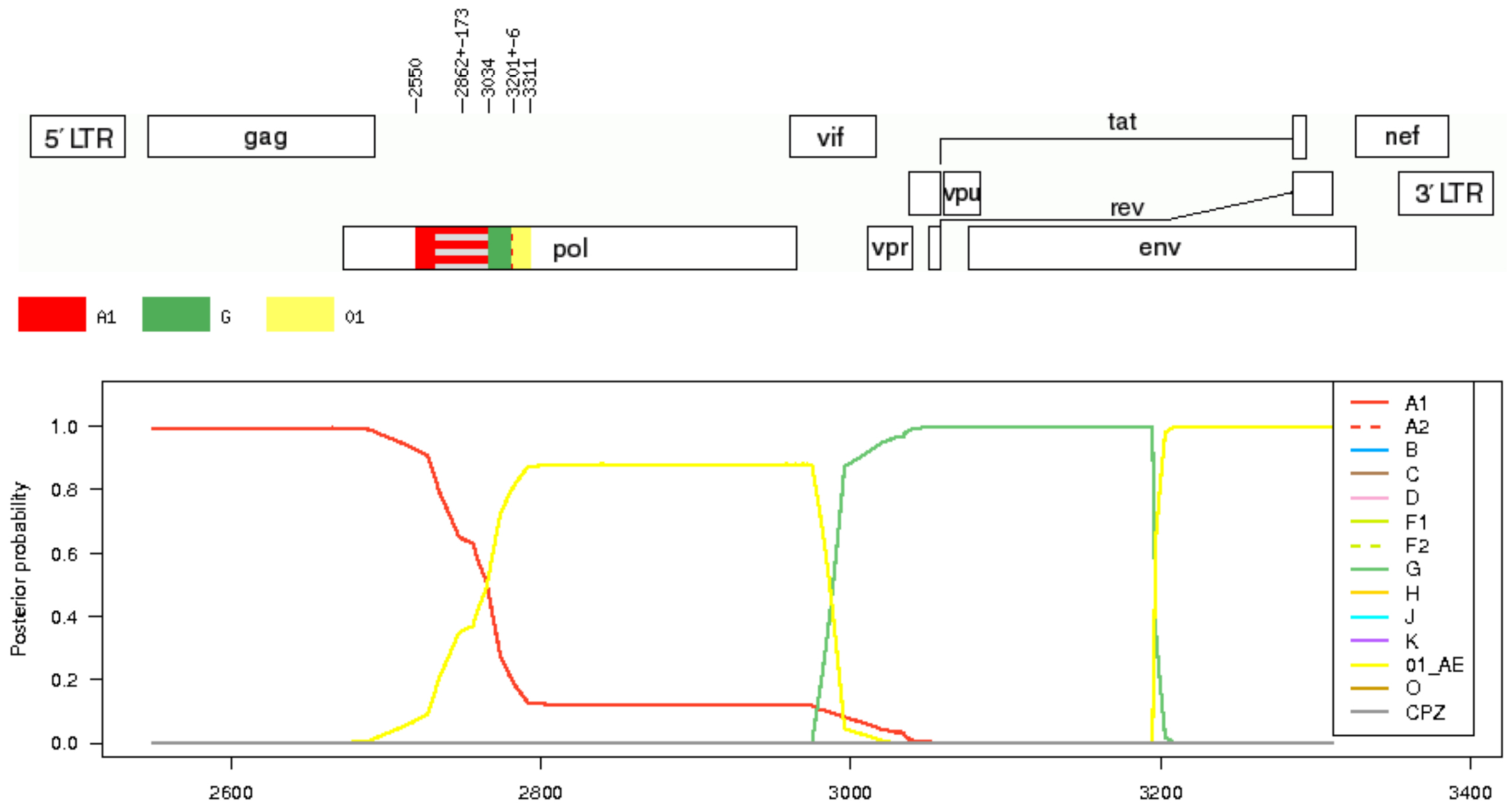

C IDU11 RT RNA

Fragment Start Position	Uncertainty Region Start - End	Break Point Interval Start - End	Fragment End position	Fragment Subtype
2550	2550 – 2888	-	2888	Subtype B
2889	2889 – 2909	-	3311	CRF01_AE

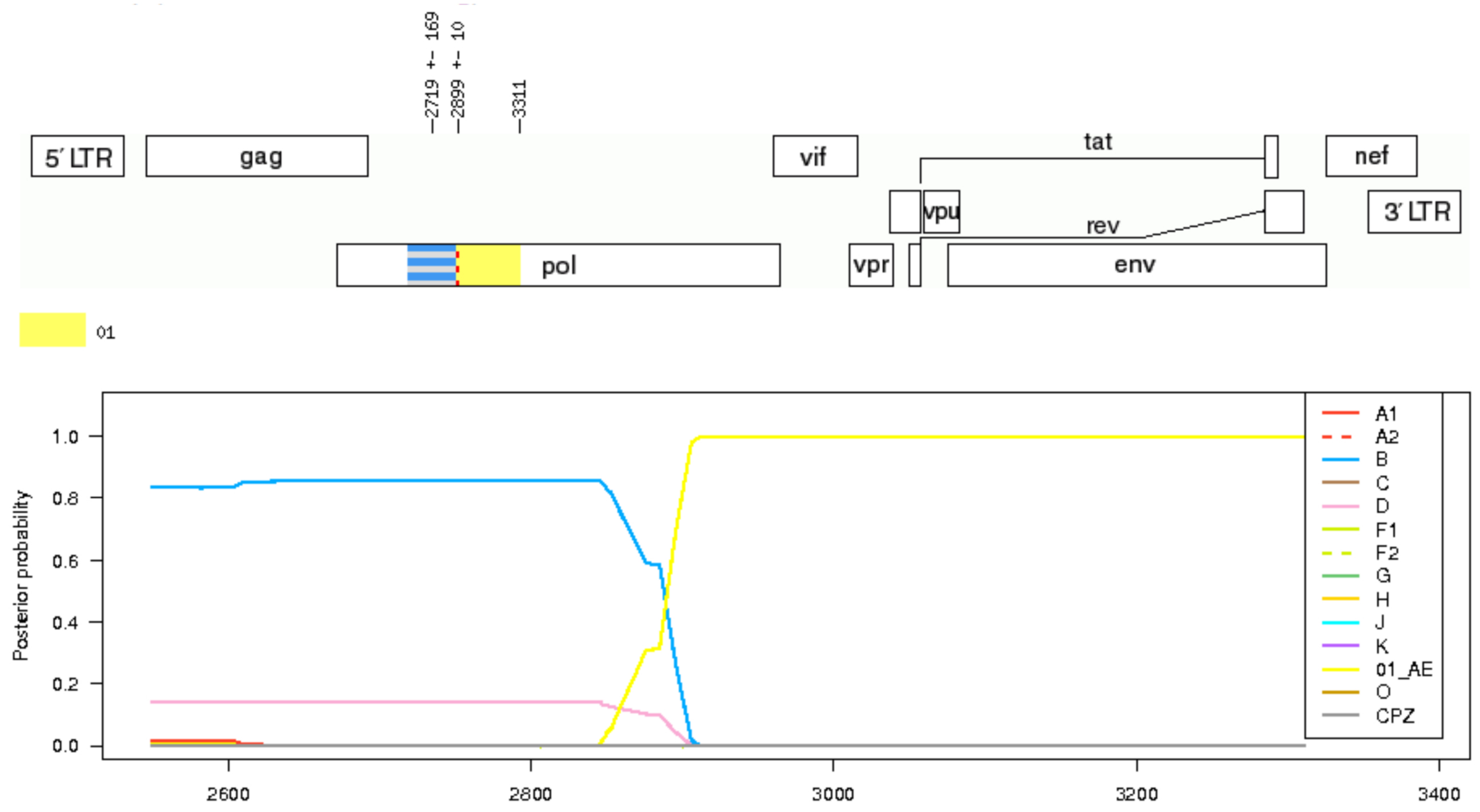

Supplement: Additional file 2: — Subtyping analysis of the recombinant HIV-1 strains using jpHMM A-C). jpHMM results of SM11, PJ121 and IDU11. Numbers in this figure are based on HXB2 numbering. Posterior probability values are indicated on the y axis and nucleotide positions based on the HXB2 sequence are shown on the x axis. [file 12981_2015_46_MOESM2_ESM.pdf]
