# Supplementary material for: HIV-1 transmitted drug resistance mutations among antiretroviral therapy-Naïve individuals in Surabaya, Indonesia
Source: AIDS Res Ther. 2015 Feb 22;12:5. doi: 10.1186/s12981-015-0046-y (PMC4336490; doi:10.1186/s12981-015-0046-y)
Supplement: Additional file 3: — Primer information. [file 12981_2015_46_MOESM3_ESM.pdf]

Additional file 3

Primer information

Name	Sequence	Nucleotide position*	Usage
K-env-R1	5'-CCAATCAGGGAAGAAGCCTTG-3'	9168 to 9148	Reverse transcription
Pr-S1	5'-TTTCTTCAGAGCAGACCAGAGCCAACAGC-3'	2131 to 2159	PR first PCR sense
RT-A1	5'-GCTTTTATTTTTTCTTCTGTCAATGGCCA-3'	2647 to 2619	PR first PCR antisense
ProCT-S-PvuI	5'-GCCGATCGCGATCGACAAGGAACTGTATCCTTTAGCTTCCCTCAGATC-3'	2220 to 2261	PR nested PCR sense
RT-CA2-AS-ClaI	5'-GTATCGATAGGACTAATGGGAAAATTTA-3'	2569 to 2542	PR nested PCR antisense
RT1L	5'-ATGATAGGGGGAATTGGAGGTTT-3'	2388 to 2410	RT first PCR sense
GPR2M	5'-GGACTACAGTCYACTTGTCCATG-3'	4402 to 4380	RT first PCR antisense
RT7L	5'-GACCTACACCTGTCAACATAATTGG-3'	2485 to 2509	RT nested PCR sense
GPR3L	5'-TTAAAATCACTARCCATTGYTCTCC-3'	4309 to 4285	RT nested PCR antisense

* Nucleotide position was based on a HIV-1 reference strain, HXB2 (GenBank accession no. K03455).
